# MRFGRO: a hybrid meta-heuristic feature selection method for screening COVID-19 using deep features

**DOI:** 10.1038/s41598-021-02731-z

**Published:** 2021-12-15

**Authors:** Arijit Dey, Soham Chattopadhyay, Pawan Kumar Singh, Ali Ahmadian, Massimiliano Ferrara, Norazak Senu, Ram Sarkar

**Affiliations:** 1 Department of Computer Science and Engineering, Maulana Abul Kalam Azad University of Technology, Kolkata, West Bengal 700064 India; 2 Department of Electrical Engineering, Jadavpur University, 188, Raja S. C. Mallick Road, Kolkata, West Bengal 700032 India; 3 Department of Information Technology, Jadavpur University, Jadavpur University Second Campus, Plot No. 8, Salt Lake Bypass, LB Block, Sector III, Salt Lake City, Kolkata, West Bengal 700106 India; 4 Institute of IR 4.0, The National University of Malaysia, 43600 Bangi, Malaysia; 5 Department of Mathematics, Near East University, Nicosia, TRNC, Mersin 10, Turkey; 6 Department of Management and Technology, ICRIOS - The Invernizzi Centre for Research in Innovation, Organization, Strategy and Entrepreneurship, Bocconi University, Via Sarfatti, 25, Milan, MI 20136 Italy; 7 Institute for Mathematical Research, Universiti Putra Malaysia (UPM), 43400 Selangor, Malaysia; 8 Department of Computer Science and Engineering, Jadavpur University, 188, Raja S.C. Mallick Road, Kolkata, West Bengal 700032 India

**Keywords:** Infectious diseases, Computer science, Scientific data, Software

## Abstract

COVID-19 is a respiratory disease that causes infection in both lungs and the upper respiratory tract. The World Health Organization (WHO) has declared it a global pandemic because of its rapid spread across the globe. The most common way for COVID-19 diagnosis is real-time reverse transcription-polymerase chain reaction (RT-PCR) which takes a significant amount of time to get the result. Computer based medical image analysis is more beneficial for the diagnosis of such disease as it can give better results in less time. Computed Tomography (CT) scans are used to monitor lung diseases including COVID-19. In this work, a hybrid model for COVID-19 detection has developed which has two key stages. In the first stage, we have fine-tuned the parameters of the pre-trained convolutional neural networks (CNNs) to extract some features from the COVID-19 affected lungs. As pre-trained CNNs, we have used two standard CNNs namely, GoogleNet and ResNet18. Then, we have proposed a hybrid meta-heuristic feature selection (FS) algorithm, named as Manta Ray Foraging based Golden Ratio Optimizer (MRFGRO) to select the most significant feature subset. The proposed model is implemented over three publicly available datasets, namely, COVID-CT dataset, SARS-COV-2 dataset, and MOSMED dataset, and attains state-of-the-art classification accuracies of 99.15%, 99.42% and 95.57% respectively. Obtained results confirm that the proposed approach is quite efficient when compared to the local texture descriptors used for COVID-19 detection from chest CT-scan images.

## Introduction

The first case of the COVID-19 was witnessed in the city of Wuhan, China in December 2019. It has since spread across the globe leading to an ongoing pandemic. It spreads through a respiratory path while a person gets close to an infected person. As there are no such medicines for this till date, early detection is very much required. The common way of COVID-19 detection is real-time reverse transcription-polymerase chain reaction (RT-PCR), but it has a low rate of detection accuracy (around 60–70%) and even after getting the negative results radiological traces are found in the chest computed tomography (CT) scan images^1^. Moreover, it takes almost a day to give the results. On the other hand, the CT scan is a non-invasive, painless process that allows radiologists to monitor cross-sectional levels of lungs by using a rotating X-ray beam. Many diseases such as lung cancer, infiltration, hernia, pneumonia, etc. can be diagnosed by analyzing the CT scans through computer-aided systems. Moreover, X-ray images are less portable and less ionized but the CT scan images are more preferable because it gives the more comprehensive architecture of lung’s air sacs and gives accurate estimation to predict the size, shape and the structure of the lung^2^. In this paper, we have used CT scan images to detect COVID-19 using a low computational model by reducing the dimension of the feature space using meta-heuristic approach.

**Figure 1:**
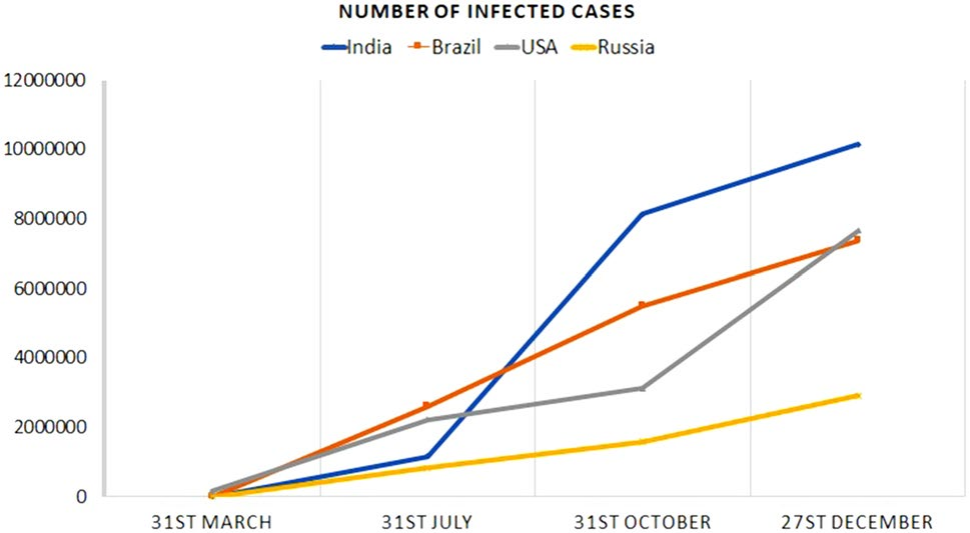
Increasing number of COVID cases in some countries. The data have been collected from the official website of WHO^6^.

The actual origin of the coronavirus is not discovered yet^3^. Scientists estimate that the origin of this virus can be zoonotic natured animals. However, genetic analysis has confirmed that it has 96% identical genome level with the coronavirus samples of bat (BatCov RaTG13)^4^. The first infected person was noticed in Hubei market, Wuhan, China, and eventually it affected the other people^5^. Globally, 76.9 million people are infected till 21 December 2020. Almost, every country has been affected more or less. Somehow, China and a few other countries have managed to control this pandemic in their countries. The USA is the most affected with 17.9 million confirmed cases, and India comes second in this list. Unfortunately, 1.7 million people all over the world lost their lives due to COVID-19. In the mid of March, Italy was the most affected having the highest number of casualties due to COVID-19^6^. Figure 1 shows an increasing number of cases in a few countries over the last 10 months.

The virus affects the lungs of an infected person. A study shows that lungs get puffed up and shadowy patches are noticed in the CT scan images of an infected person, the phenomenon is known as Ground Glass Opacity^7^. Due to its communicable nature, the spread of the virus is much faster than its detection rate. The symptoms are quite similar to chronic pneumonia as the lungs get inflamed.

In this paper, we have proposed a model to detect COVID-19 from chest CT-scans where both machine learning and deep learning approaches are used. Deep learning models learn features automatically by themselves. Whereas, machine learning approaches can give results with a low computational cost. For image processing tasks, there are different types of traditional feature extraction techniques but here we have used deep features from five pre-trained convolutional neural networks (CNNs) which are GoogLeNet^8^, ResNet18^9^, ResNet152^9^, VGG19^10^ and VGG16^10^. We have concatenated all the features and get a high dimensional feature vector. As we extract features from different CNNs, many redundant features may be included in the concatenated feature vector. To remove the redundancy and increase the accuracy of the model, we have developed a hybrid meta-heuristic approach for feature selection. Now the question arises that why we need a hybrid feature selection model? *Nofreelunch*^11^ theorem emphasizes that there is no such algorithm that can solve every optimization problem. Besides, the Manta ray foraging optimizer (MRFO) has a good exploration property and the Golden ratio optimizer (GRO) can explore closer to the local minimum. The hybridization of MRFO and GRO helps to balance between good exploration and exploitation. The proposed hybrid algorithm is known as Manta Ray Foraging based Golden Ratio Optimizer (MRFGRO). The contributions of the paper are listed below.

- We have fine-tuned the parameters of CNNs and extracted features from different pre-trained CNNs (GooGLeNet, ResNet18, ResNet152, VGG19, and VGG16) and compare each combination to get the better performing model. The combination of GoogLeNet and ResNet gives the best result among all other combinations (detailed discussion in “Deep feature extraction” section)
- Though individual CNN model has less redundant features, we have proposed a hybrid meta-heuristic approach MRFGRO to reduce the overall feature dimension and increase the model’s overall classification accuracy. That is, the MRFGRO algorithm focuses on reducing the dimension of feature space and which further leads in achieving faster and better classification results. We have compared the results with other optimization algorithms and achieved better results from them (detailed discussion in “Comparison with other optimization algorithms” section).
- We have evaluated our model on three publicly available datasets namely, COVID-CT, Sars-CoV-2, and MosMed, and achieved accuracies of 99.15%, 99.42% and 95.57% respectively.

## Literature survey

In this section, we have described some existing methods for COVID-19 detection using machine learning and deep learning models. Disease detection from CT scan images with various computer-aided systems have started in the end of the twentieth century. Many chronic disease detection become very easy with deep learning and machine learning based models.

Different machine learning and deep learning models have been proposed to diagnose different lung diseases including COVID-19 and chronic pneumonia. The basic constraint for COVID-19 detection using medical images is the lack of data. That is the reason, Waheed et al.^12^ have proposed an Auxiliary Classifier Generative Adversarial Network (ACGAN) that generates the number of images that can help to increase the performance of CNNs. However, Horry et al. have used a transfer learning model on different multimodal COVID datasets^13^. Sabanci et al. have introduced a conjugated system with a pre-trained CNN to a Bidirectional Long Short-Term Memories (BiLSTM) to emphasize the temporal features^14^. Matteo Polsinelli has proposed a light CNN namely SqueezeNet and implemented on the dataset developed by Zhao et al. and gets an accuracy of 83.3%^15^. Wang et al.^16^ have proposed a deep CNN and trained it with 13,975 X-ray images and get an accuracy of 98.9%. In another research, Ying et al.^17^ have introduced a DRE-Net to classify COVID and healthy patients using chest CT-scan images and achieved an accuracy of 86%. Also, Ozturk et al. have proposed a 17 layer CNN named DarkCovidNet. This model has got an accuracy of 87.02% for three-class classification and 98.08% for two-class classification. Moreover, Rajarshi et al.^18^ have developed a model which extracts deep features from various CNNs and thereafter the optimal feature subset selection has been done using Harris Hawks optimisation with Simulated Annealing algorithm. The proposed method has been evaluated on SARS-COV-2 CT-Scan dataset and their obtained accuracy was 98.85%. Table 1 shows some further works on different models for automated COVID detection using medical image analysis.

**Table 1:** Summarization of previous works reported for COVID-19 detection.

Work ref.	Method	Dataset	Obtained accuracy
Shibly et al.^19^	Used faster R-CNN	COVIDx dataset	97.65%
Zheng et al.^20^	UNet+3D network	Own dataset	90.8%
Jaiswal et al.^21^	DenseNet 201	SARS-Cov-2 dataset	96.25%
Soares et al.^22^	xDNN	SARS-Cov-2 dataset	97.38%
Panwar et al.^23^	Gradient-weighted class activation mapping (Grad-CAM)	Cohen dataset	97.08%
Kundu et al.^24^	Fuzzy rank-based fusion of VGG-11, Wide ResNet-50-2, and Inception v3	SARS-COV-2 dataset and Harvard Dataverse chest CT dataset	98.93% and 98.80% (respectively on SARS-COV-2 and Harvard Dataverse chest CT datasets)

From the literature survey, it is understood that most of the researchers have relied on different deep learning models for the detection of COVID-19 from medical images^25^. So, from the above discussion, we can say that different CNN based models have different capabilities of feature extraction from the input images. However, if we concatenate the feature vectors obtained from those models, then it would become a high dimensional feature vector which, in turn, needs more storage and a huge amount of time to train a model. Here lies the requirement of an FS model that can eliminate the redundant features from the extracted deep feature set. Meta-heuristic^26^ approaches are quite popular to manage this task. In recent times, different feature selection techniques have been introduced. Although, we have mentioned different optimization algorithms in this paper. Researchers have found that a single optimization algorithm might fail to deal with every problem^11^. Some of recent times hybrid optimization algorithms are: cooperative Genetic Algorithm (CGA)^27^, Late Acceptance Hill-Climbing (BBA-LAHC)^28^, hybridization of Mayfly algorithm (MA) and HS named as MA-HS algorithm^29^, hybridization of GA with PSO and Ant Colony Optimization (ACO) algorithm^30^, clustering-based equilibrium and ant colony optimization (EOAS)^31^. Keeping the above facts in mind, in the proposed work, we have proposed a hybrid meta-heuristic FS algorithm, called MRFGRO, which reduces the feature dimension of the features obtained from the deep learning models when applied over chest CT scan images to detect the COVID-19.

## Materials and methods

In this section, the workflow of the proposed approach for COVID-19 detection has been discussed successively. The entire work is divided into different subsections that include: (A) dataset description, (B) deep feature extraction, and (C) feature selection.

### Dataset description

In this paper, we have evaluated our model on three publicly available datasets which are briefly described below.

#### COVID-CT dataset

The covid-CT dataset is developed by Jhao et al.^32^. As the name suggests, this dataset consists of chest CT-scan images with 349 confirmed COVID-19 cases and 397 healthy cases. In this research framework, all images are resized to $224 \times 224 \times 3$ and are normalized before feeding them to the deep learning frameworks for feature extraction. During the training process of deep neural networks, as the dataset is very small, the size of the dataset is augmented by a rotation of $50^{\circ }$, a slant-angle of $0.5^{\circ }$, as well as by enabling horizontal and vertical flipping.

#### SARS-Cov-2 dataset

SARS-Cov-2 CT-scan dataset is developed by Soares et al.^22^. This dataset contains 2492 chest CT-scan images, out of which 1262 are COVID-19 positive and the rest 1230 images are of a healthy subject. Similar to the previous dataset, the images are also resized to $224 \times 224 \times 3$ and during training, data augmentation techniques are applied with $25^{\circ }$ of rotation and horizontal flip.

#### MOSMED dataset

This dataset^33^ consists of CT-scan images of 1110 patients, divided into five different classes. The classes are as follows:

- *CT[0]* Normal lung tissue with no sign of viral pneumonia.
- *CT[1]* Multiple ground-glass opacity is noticed and lung parenchyma is involved 25%.
- *CT[2]* Multiple ground glass opacity is noticed and lung parenchyma is involved 25–50%.
- *CT[3]* Multiple ground-glass opacity is noticed and lung parenchyma is involved 50–75%.
- *CT[4]* Multiple ground-glass opacity is diffused and lung parenchyma is involved more than 75%.

### Deep feature extraction

Sometimes it is difficult to design a competent feature vector using conventional feature engineering techniques when the underlying dataset is very complex. Moreover, it is found that such a feature vector designed for a particular dataset may not perform well when applied to other datasets. Hence, in this research work, we have focused on extracting deep features using pre-trained CNN models. For deep feature extraction, we have considered five standard pre-trained CNNs such as GoogLeNet^8^, ResNet18^9^, ResNet152^9^, VGG19^10^ and VGG16^10^. All of the pre-trained CNNs are fine-tuned on the datasets for 30 epochs of training. For all cases, cross-entropy loss^34^ has been optimized by Adam optimizer^35^ with learning rate and momentum of 0.0009 and 0.85 respectively. After 30 epochs of training, the weights of the epoch which achieves the minimum loss have been loaded and the model is set to its evaluation mode. Thereafter, both the training and testing images are passed through the model, and the features from the last layer have been extracted. This is how deep feature extraction has been performed in this study. The numbers of deep features extracted using different CNNs are shown in Table 2.

**Table 2:** Number of features obtained from different deep learning models when applied over COVID-19 datasets.

Pre-trained CNN	Number of features extracted
ResNet18	512
ResNet152	2048
VGG16	25,088
VGG19	25,088

Also, to evaluate deep features obtained from different CNNs together, we have tested the combinations of different CNNs by fusing the feature sets and evaluated through our proposed MRFGRO algorithm for FS. In the fusing process, the features from different CNNs are concatenated together to form the final feature vector. Suppose from CNN1 and CNN2, the extracted features are *f*1 and *f*2, and suppose, after the fusion function (*F*(.)), the final feature vector becomes *f*. Therefore

1 $$\begin{aligned} {f = F(f1, f2)}. \end{aligned}$$

**Figure 2:**
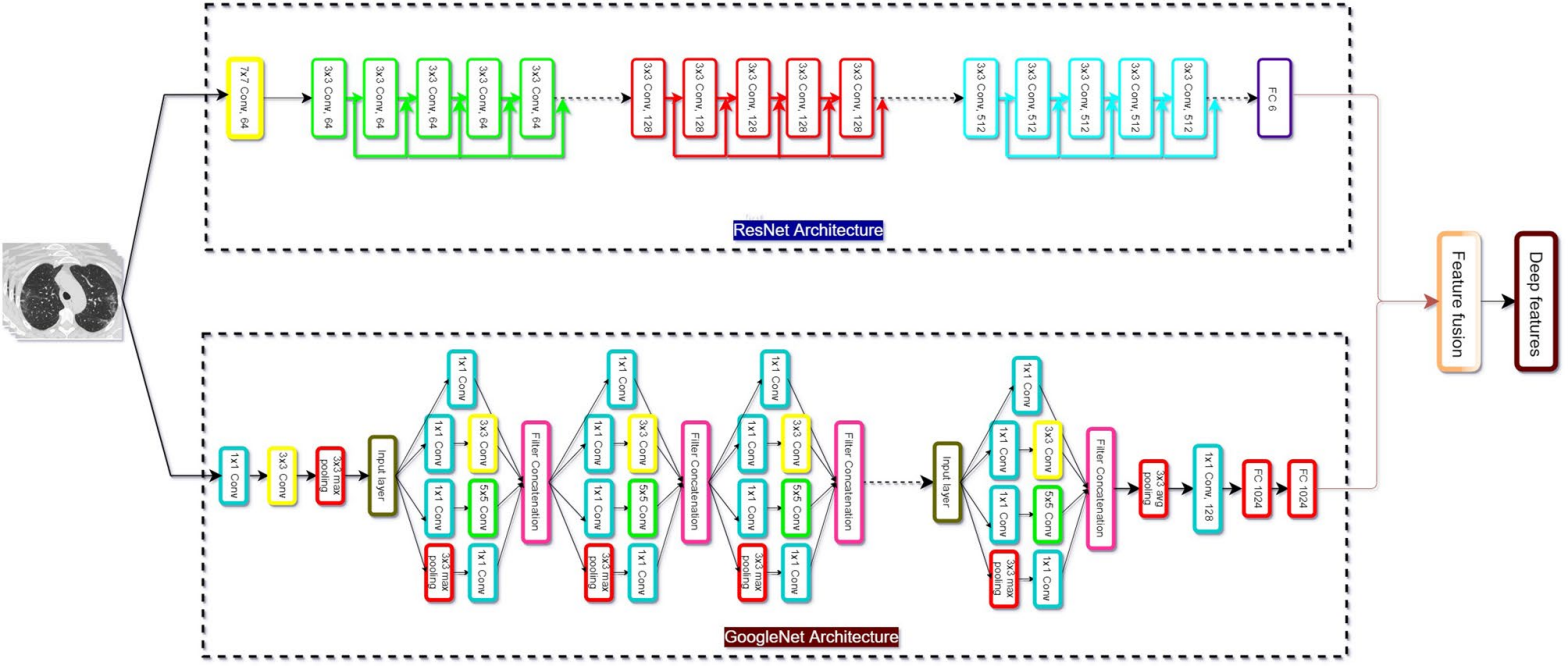
Illustration of the work flow of deep features extraction from GoogLeNet and ResNet18 architectures. The input CT-scan images are taken from CARS-Cov-2 CT-scan dataset^22^.

Then, the number of features in *f* would be the summation of the number of features of the feature set of each CNN.

2 $$\begin{aligned} {N_f = \sum _{i=1}^n N_{fi}}, \end{aligned}$$

where $N_f$ is the number of features the fused feature set has and $N_{fi}$ is the number of features in the $i\mathrm{{{th}}}$ deep feature set. The results are obtained from different features from different nets and their combinations are provided in “Results and discussion” section. Additionally, the representational diagram depicting deep feature extraction process is given by Fig. 2.

### Feature selection model

We have extracted features from different CNNs and concatenated them in various combinations. As a result, the size of the feature set becomes very large. Therefore, there remains a chance that such larger sized feature vector might overfit the classifiers and there may be some redundant features. So, to address this issue, we design a FS algorithm that can produce a more prolific feature subset out of the entire feature set. In doing so, we propose a new hybrid meta-heuristic FS algorithm known as MRFGRO algorithm by hybridizing MRFO with GRO. One of the main limitations of the FS model is the premature convergence and the get stuck at the local minimum. However, a hybrid model can help to balance between exploration and exploitation, so that the problem of premature convergence can be overcome. The working mechanism of each candidate optimization algorithm and their hybridization procedure are discussed in the next subsection.

#### Manta ray foraging optimizer

MRFO^36^is one of the two optimization algorithms which we have chosen to produce our hybrid FS model. MRFO is based on the foraging properties which manta rays use to haunt their prey. Three different foraging strategies have been used in the algorithm, which are chain foraging, cyclone foraging, and somersault foraging. In the first type of foraging technique, manta rays aim to achieve a high level of concentration to catch their prey plankton. Therefore, they form a foraging chain, while each manta ray is after the prey and their position is being updated over the iterations. The mathematical expression of chain foraging is as followed:

3 $$\begin{aligned} p_j^{(n+1)}= \left\{ \begin{array}{ll} p_j^n+d(p_{best}^n-p_j^n)+\beta (p_{best}^n-p_j^n)&{} j=1 \\ p_j^n+d(p_{j-1}^n-p_j^n)+\beta (p_{best}^n-p_j^n)&{} j=2,\ldots,N \end{array} \right., \end{aligned}$$

at iteration *n*, the position of $j\mathrm{{{th}}}$ manta ray is given by $p_j^n$ and, *d*, *N* and $p_{best}^n$ are a random vector, number of manta rays and the best solution respectively. The weighting coefficient $\beta$ is given by

4 $$\begin{aligned} 2 \times d \times \sqrt{|log(d)|}. \end{aligned}$$

Manta rays start forming chain in a combined manner and swim towards the prey following a spiral path, after being cognizant about the exact position of the plankton. In cyclone foraging, in addition to spiral motion, each manta ray is one step ahead towards its prior one, and thus a cyclonic motion in formed. The cyclonic foraging can be expressed in terms of two perpendicular components, which are given as follows:

5 $$\begin{aligned} X_j^{n+1}= & {} X_{best}+d(X_{j-1}^n-X_j^n)+e^{a\omega }cos(2\pi \omega )(X_{best}-X_j^n), \end{aligned}$$

6 $$\begin{aligned} Y_j^{n+1}= & {} Y_{best}+d(Y_{j-1}^n-Y_j^n)+e^{a\omega }sin(2\pi \omega )(Y_{best}-Y_j^n), \end{aligned}$$

where $\omega$ is a random number. Now similar to chain foraging, the position and movement of cyclone foraging towards the minimum can be expressed as given below:

7 $$\begin{aligned} p_j^{(n+1)}=\left\{ \begin{array}{ll} p_{best}+d(p_{best}^n-p_j^n)+\gamma (p_{best}^n-p_j^n)&{} j=1 \\ p_{best}+d(p_{j-1}^n-p_j^n)+\gamma (p_{best}^n-p_j^n)&{} j=2,....,N \end{array} \right.. \end{aligned}$$

Here, also $\gamma$ is a weighting factor with the expression

8 $$\begin{aligned} \gamma = 2e^{d_1\left( \frac{I-n+1}{I}\right) }sin(2\pi d_1), \end{aligned}$$

where *I* is the maximum iteration and *d*1 is a random number. Since manta rays search for the prey from their reference positions, cyclone foraging has good exploitation towards the search of the best solution. In addition, cyclone foraging process exert forces to each manta ray or candidate solution to search for new best solution which remains far from the current best. That’s how exploitation is enhanced here. This is performed by assigning a random position in the search space,

9 $$\begin{aligned} p_{rand} = lb+d(lb-ub), \end{aligned}$$

and

10 $$\begin{aligned} p_j^{(n+1)}=\left\{ \begin{array}{ll} p_{rand}+d(p_{rand}^n-p_j^n)+\gamma (p_{rand}^n-p_j^n)&{} j=1 \\ \\ p_{rand}+d(p_{j-1}^n-p_j^n)+\gamma (p_{rand}^n-p_j^n)&{} j=2,....,N \end{array} \right., \end{aligned}$$

where, $p_{rand}$ is the randomly assigned position and *lb*, *ub* are the lower bound and the upper bound of problem variables respectively.

The final stage of this MRFO is the somersault foraging, where the food is chased as a hinge. In this type of foraging, each manta ray tumbles around the hinge for a new position. The motion can be expressed as

11 $$\begin{aligned} p_j^{(n+1)} = p_j^{(n)}+S \times (d_2p_{best}-d_3p_j^{(n)}) , \quad j=1,2,....,N, \end{aligned}$$

where, *S* is the somersault foraging factor, and $d_2, d_3$ are random numbers. This is the last phase and here the distances between the emerging solutions and the global minimum get reduced and converge to optimal solution. Eventually this foraging reduces adaptively over the iterations. This is how MRFO approaches the optimal solution by developing a mimic to the haunting process of manta ray fishes.

#### Golden ratio optimization

There are various physical phenomena which form a fixed ratio known as golden ratio^37^. Fibonacci first introduced the term golden ratio. He defined a series called Fibonacci series, which is basically an infinite series where the $k\mathrm{{{th}}}$ is the sum of $(k-1)\mathrm{{{th}}}$ and $(k-2)\mathrm{{{th}}}$ terms. The ratio of any two consecutive terms in the series is always a fixed number 1.618, this number is named as golden ratio. This is the key idea of the GRO algorithm. $k\mathrm{{{th}}}$ Fibonacci number can be obtained from the following equation.

12 $$\begin{aligned} F\left( k\right) =GR\ .\frac{\left( \ \gamma ^k-\left( 1-\ \gamma ^{-k}\right) \right) }{\sqrt{5}}\ \ \ \ where\ GR\ =\ 1.618. \end{aligned}$$

Similar to other wrapper based FS algorithms, here also initial population is generated. In GRO algorithm, the candidate solutions are considered as vectors. These vectors have certain magnitudes and directions as well. The directions and the magnitudes of these vectors are updated over the iterations and moved towards the global minimum. Initially, the mean value of the population is chosen and the fitness of each candidate solution is calculated. Thereafter, each candidate solution of the population is compared to the mean solution of the population. Now, if the fitness of the mean solution is more than the worst solution, then the worst solution is replaced by the mean solution. This process is carried out in an iterative manner by updating the population in each iteration. Again the worst solution within the updated population is calculated and the above steps are repeated. Thus the vectors of the population get converged towards the minimum.

13 $$\begin{aligned} {D}_{best}> & {} {D}_{medium}>\ {D}_{worst}, \end{aligned}$$

14 $$\begin{aligned} W_{t\ }= & {} W_{medium\ }-\ W_{worst\ }. \end{aligned}$$

**Figure 3:**
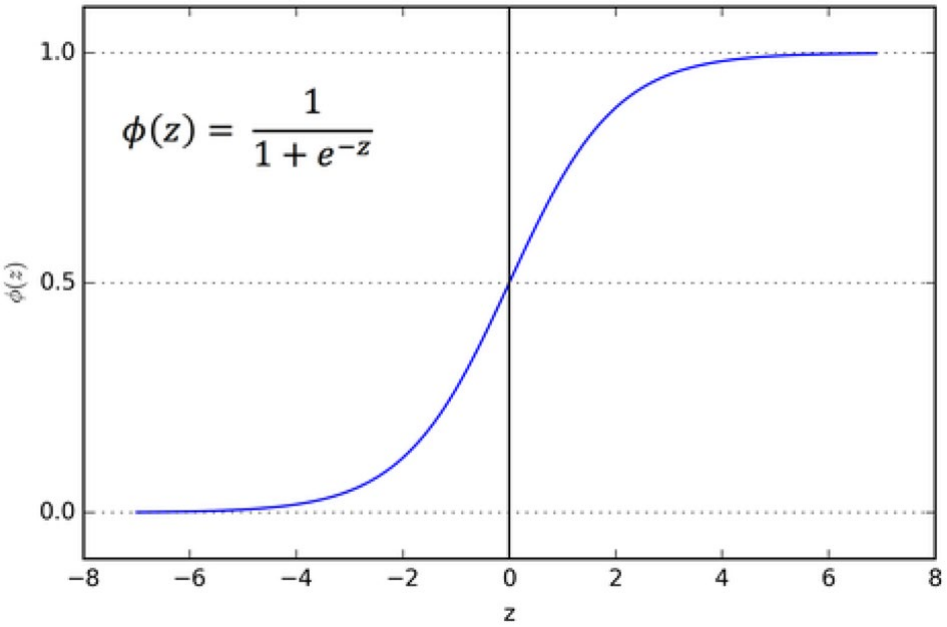
Graphical representation of sigmoid function.

Equations (13) and  (14) represent the absolute and direction of the movement of the solution vectors during the search of the global minimum. Now to enhance the exploration of the algorithm, each time during the upgrade of the population a random movement is added to the population. This improves the search of the minimum in the entire solution space rather than in a particular region. The equation representing this phenomena is given by Eq. (15).

15 $$\begin{aligned} W_{new }=\left( 1-{D}_{t}\right) W_{best\ }+rand \cdot W_{t\ } \cdot {D}_{t}. \end{aligned}$$

This is how solutions are updated and if the boundary condition is reached, the new solutions replace the old solutions in the population.

### Proposed algorithm

**Figure 4:**
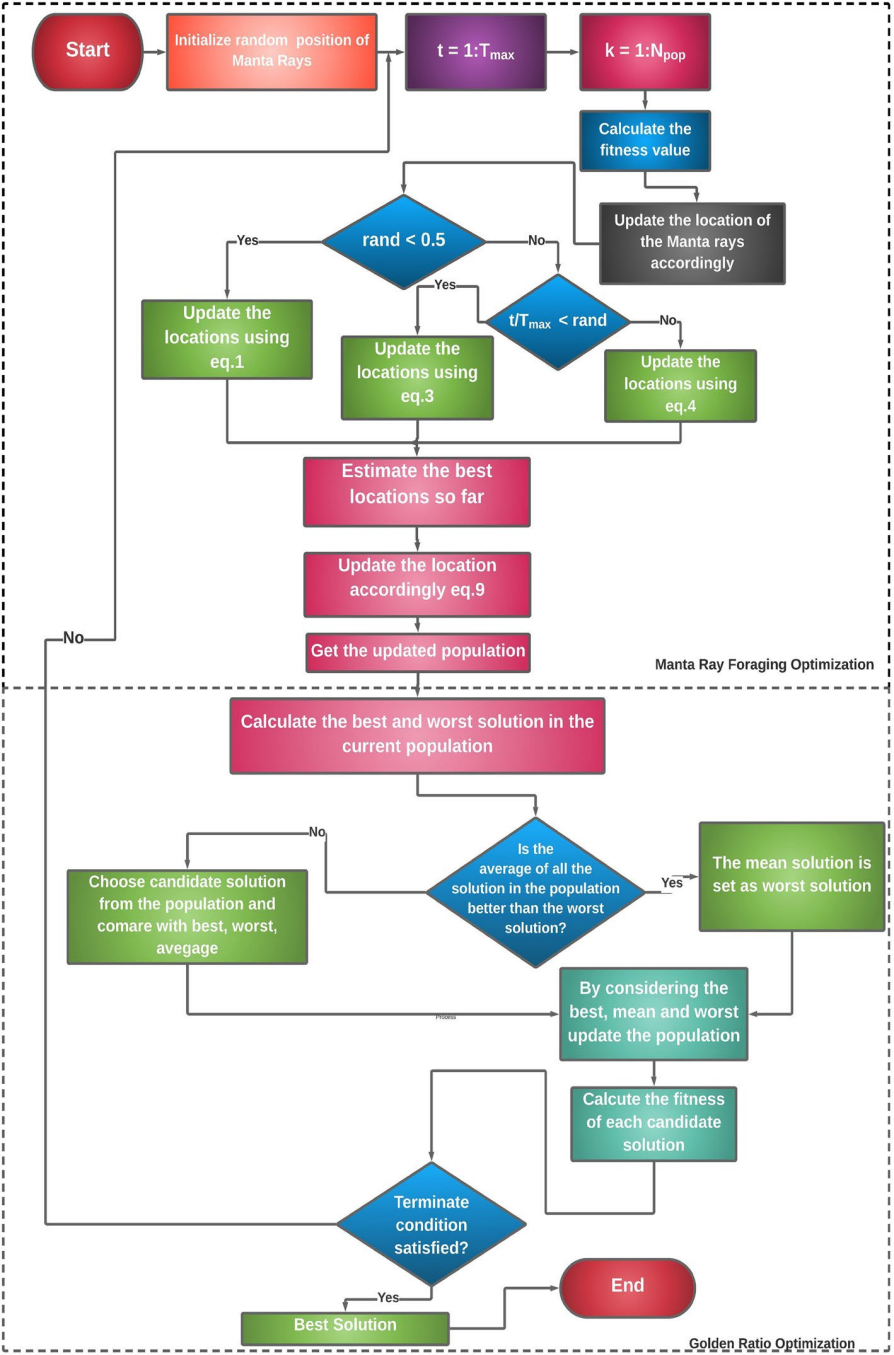
Proposed workflow of our proposed MRFGRO algorithm.

The proposed MRFGRO (see Fig. 4) algorithm is the hybridized form of MRFO and GRO. The main motive of this hybridization is to overcome the drawback of the parent algorithms. The extracted feature set is represented by 0’s and 1’s, where 1 represents the feature to be selected and 0 represents exactly the opposite. Again, the basic goal of FS algorithms is to reduce the number of 1’s and achieve higher accuracy accordingly. Optimization in continuous search space is quite opposite than used in the binary search space. The binary search space is considered as a hypercube and the search agents try to jump nearer the hypercube by changing the bits. Two widely used transfer functions which are applied to convert the continuous optimization problem to a binary optimization problem are S-shaped and V-shaped transfer functions. S-shaped function is represented by Eq. (17). However, in this paper, we have used S-shaped transfer function.

16 $$\begin{aligned} fitness (x) = \omega \cdot A_{classifier} + (1 - \omega )(1 - |\theta /\theta ^{'}|). \end{aligned}$$

#### Transfer function

The role of the transfer function to convert the feature set into series of 0’s and 1’s to perform the final training of the sample. For this purpose we have used signoid function for binarization. As we know, the output of the sigmoid function ranges between 0 and 1. Eq. (17) refers to the sigmoid function. Figure 3 shows the graphical representation of sigmoid function.

17 $$\begin{aligned} t_{s}(x) = 1/1+e^{-x}. \end{aligned}$$

Our proposed algorithm has the following steps:

- *Step 1* Fine-tune the control parameters of MRFO: population size ($N_{pop}$), maximum number of iterations ($T_{max}$) and somersault factor ($S_{f}$).
- *Step 2* Initialize the randomly generated positions of the manta rays.
- *Step 3* Calculate fitness of every solution of the generated population using Eq. (16) and update the location of the manta rays accordingly.
- *Step 4* The exploration and exportation are maintained by $t/T_{max}$ and if the fitness value is less than the rand, exploitation takes place, otherwise exploration is executed. If the value of $rand > 0.5$, the positions of the manta rays get updated using Eq. (3). Further, if the value of $t/T_{max}$ is less than rand, then positions get updated accordingly Eq. (5) else using Eq. (6).
- *Step 5* Estimate the fitness value and update the position. Then calculate the best and worst solutions of the current solution and average as well.
- *Step 6* Then best, worst and average are compared with the current candidate solution and if the terminate condition is satisfied, the optimization stops there and gives best solution as an output, otherwise it goes to step 2.

### Overview of the classifiers used

For calculation of fitness function, we have chosen three different state-of-the-art classifiers, such as SVM^38^, MLP^39^ and ELM^40^. In the proposed algorithm the SVM classifier is evaluated with kernel function *’rbf’*, and the hidden layers of MLP and ELM are fixed to 5. The regularisation parameter *’C’* of SVM has a value of 5000.

### Statement

All experiments and methods were carried out in accordance with relevant guidelines and regulations.

## Results and discussion

In this section, we report the experimental results on the three COVID-19 detection datasets, brief descriptions of which are already given in the previous section. The experimentation include the results obtained by different machine learning classifiers used for fitness calculation of MRFGRO algorithm, loss plots and accuracy plots of different deep learning models, comparison of MRFGRO algorithm with other FS algorithms, hyperparameters tuning, and so on. At the end, we conclude this section by giving comparative studies of the proposed method of COVID-19 detection with several state-of-the-art techniques.

For the evaluation purpose, we have used four standard metrics, which are Accuracy, Precision, Recall, and F1 score. All these metrics have been taken into consideration to evaluate the proposed model more generally as well as to handle the class imbalance issue. These evaluation metrics are dependant on some elementary measures, which are true positive (TP), true negative (TN), false positive (FP), and false negative (FN). The mathematical expressions for calculating aforementioned metrics based on TP, TN, FP, and FN values are given below:

- Accuracy:
  18 $$\begin{aligned} \frac{TP+TN}{TP+TN+FP+FN}, \end{aligned}$$
- Precision:
  19 $$\begin{aligned} \frac{TP}{FP+TP}, \end{aligned}$$
- Recall:
  20 $$\begin{aligned} \frac{TP}{TP+FN}, \end{aligned}$$
- F1 Score:
  21 $$\begin{aligned} \frac{TP}{TP+\frac{1}{2}(FP+FN)}. \end{aligned}$$

### Deep features

It has already been mentioned that transfer learning has been used for deep features extraction. In doing so, we have extracted features from CT scan images using the mentioned deep learning models and have used our proposed MRFGRO algorithm for feature dimension reduction and classification. We have also evaluated our model for different combinations of concatenated deep features extracted by different deep learning models. The results of some of these models on three COVID-19 detection datasets are given by Table 3.

**Table 3:** Classification results obtained with different deep feature sets using our proposed MRFGRO algorithm.

Feature set	SARS-CoV-2 CT-scan dataset		Covid-CT dataset		MOSMED dataset	
	No. of selected features	Accuracy (%)	No. of selected features	Accuracy (%)	No. of selected features	Accuracy (%)
GoogLeNet	780	94.47	680	96.22	811	91.91
ResNet18	445	92.17	328	96.91	378	90.11
ResNet152	1119	90.99	998	94.29	1242	91.49
VGG19	12,400	87.77	9442	85.48	15,987	81.24
VGG16	17,809	85.47	14,899	86.78	12,597	81.24
ResNet18+GoogLeNet	**875**	**99.42**	**756**	**99.15**	**612**	**95.57**
ResNet152+GoogLeNet	1180	97.71	987	96.18	1001	91.23
ResNet18+VGG16	15,489	90.02	14,801	92.24	17,589	92.21
GoogLeNet+VGG19	16,029	91.19	11,549	90.42	18,900	78.48
ResNet152+VGG19	15,014	88.18	17,802	85.44	11,259	80.04
ResNet18+GoogLeNet+VGG16	9002	86.48	15,809	84.48	18,792	79.99
ResNet152+GoogLeNet +VGG19	16,891	87.62	18,722	81.19	11,589	78.48

Best results are given in Bold.

**Figure 5:**
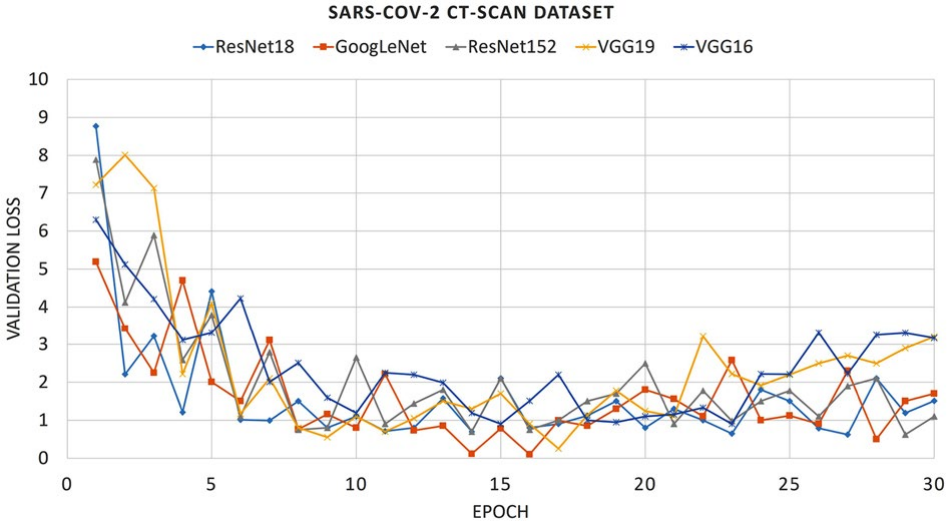
Loss plot of different deep learning models during training process on SARS-CoV-2 dataset.

**Figure 6:**
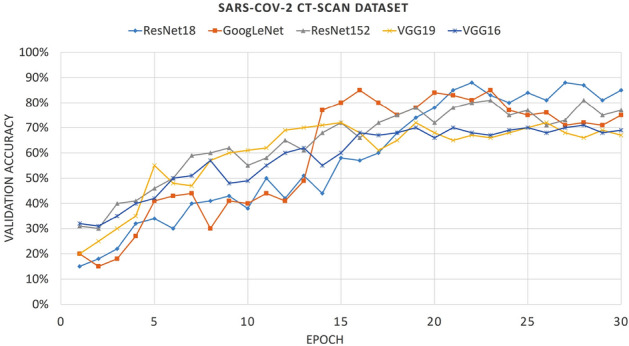
Accuracy plot of different deep learning models during training process on SARS-CoV-2 dataset.

In the previous section, it is mentioned that we have extracted deep features instead of traditional features for automatic COVID-19 detection from CT-scan images. We have trained some pre-trained networks for 30 epochs with Adam optimizer and a learning rate of 0.001. The loss function which is optimized by the optimizer is a cross-entropy loss. During training, we have used some data augmentation which is mentioned in “Dataset description” section where datasets are briefly discussed. After training, the fine-tuned weights are saved and thereafter the images are loaded, and features of the last layer are extracted. The validation loss plots and accuracy plots of all the CNNs on the SARS-CoV-2 CT-scan dataset are shown in Figs. 5 and 6. From Figs. 5 and 6, it is observed that both GoogLeNet and ResNet18 architectures converge better compare to other CNNs and the obtained accuracies are also better. The convergence loss plots of the SARS-CoV-2 CT-Scan dataset are much better as compared to COVID CT-Dataset, since the number of images in the previous one is also more. For these two datasets, the accuracies of GoogLeNet and ResNet18 happen to be much greater than that of others, but in the MOSMED dataset, all of the nets achieve comparable results. The maximum results in the SARS-CoV-2 CT-Scan dataset and COVID CT-Dataset are achieved by ResNet18, which are 92% and 90% respectively. Whereas for the MOSMED dataset GoogLeNet achieves the maximum, which is around 88%. ResNet152 performs badly on COVID CT-Dataset but gives decent result in the SARS-CoV-2 CT-Scan dataset. Both VGG16 and VGG19 thoroughly produce poor results on the SARS-CoV-2 CT-scan dataset and COVID-CT dataset but report comparable results over the MOSMED dataset.

The result obtained by combining the deep features of GoogLeNet and ResNet18 is superior to all other combinations in terms of final classification accuracy for all three datasets. For SARS-CoV-2 CT-scan dataset and MOSMED dataset, the differences in classification accuracies of different combinations are significant, whereas for COVID-CT dataset the results are much comparable. Due to very large number of features, VGG models themselves and different combinations of them fail to achieve promising results. The possible reason may be many non-informative features are generated which degrade the overall recognition accuracy. Therefore, in this case, we have combined the deep feature sets of GoogLeNet and ResNet18 models, and this is considered as our final feature set.

It is to be noted that all the results are examined by fixing the other parameters to the optimal combination. These parameters include the machine learning classifier used in calculating the fitness function, different hyperparameters of these classifiers, and various parameters of MRFGRO optimization algorithm itself.

### Calculation of fitness value

Different machine learning classifiers have been used for the calculation of the fitness value of the MRFGRO algorithm and the final classification task. The classifiers are SVM, ELM, and MLP. A brief description of these classifiers is given in the previous section. Needless to mention that the results obtained by these classifiers are numerically different from one another. The results obtained by these three classifiers upon all three datasets are reported in Table 4.

**Table 4:** Results obtained by the proposed MRFGRO algorithm using different classifiers on all three COVID-19 datasets.

Evaluation parameter	SARS-CoV-2 CT-scan dataset			COVID-CT dataset			MOSMED dataset		
	SVM (%)	MLP (%)	ELM (%)	SVM (%)	MLP (%)	ELM (%)	SVM (%)	MLP (%)	ELM (%)
Accuracy	**99.42**	97.17	98.64	**99.15**	94.44	97.98	**95.57**	90.02	92.29
Precision	97	98	**99**	**98**	92	98	**96**	91	91
Recall	**100**	97	98	**97**	95	97	**95**	91	92
F1 Score	**99**	97	98	**99**	95	97	**95**	90	90

Maximum values of accuracy, precision, recall and F1 score for each dataset are made bold.

For most of the cases of Table 4, the SVM classifier outperforms the other two in terms of accuracy as well as other evaluation metrics. In some cases, ELM classifier achieves better result than SVM classifier, however, MLP classifier have not performed so well. The results obtained by ELM classifier for SARS-CoV-2 CT-scan dataset and COVID-CT dataset, are much comparable to that of SVM classifier, but for MOSMED dataset differences are much high. Therefore, SVM classifier has been chosen for both classifications as well as fitness calculation purposes.

### Hyperparameter tuning

There are many hyperparameters in this entire framework of optimizing deep features using our proposed MRFGRO algorithm. Some are used during deep feature extraction and some are used in the proposed FS algorithm.

**Figure 7:**
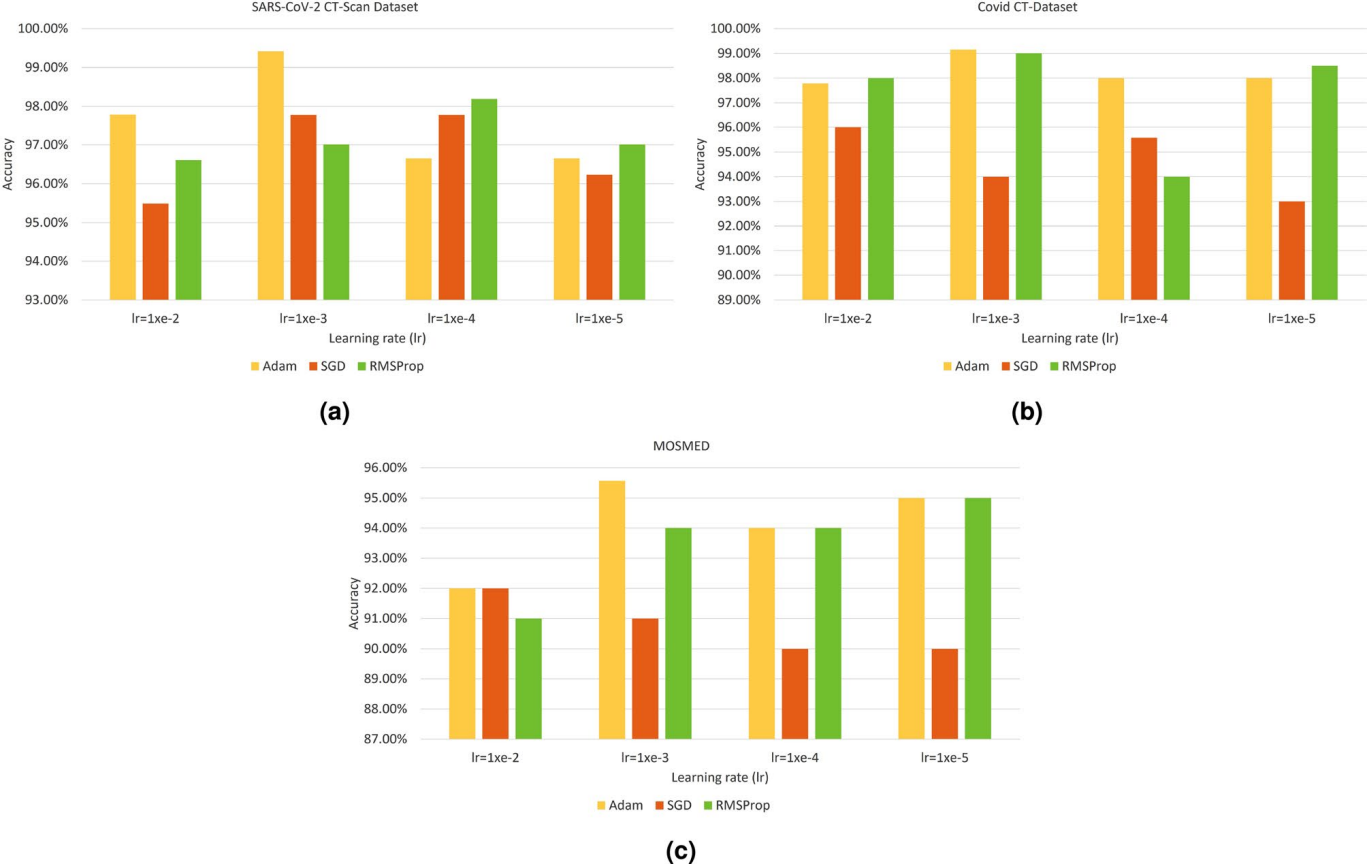
Graph showing the classification accuracies using different combinations of optimizers and learning rates on all three datasets.

The main hyperparameters of the deep learning models are the optimizer, learning rate, momentum of the optimizer, and batch size among others. In the training procedure, the optimizer and learning rate have been set to Adam and $1e^{-3}$ for all three datasets. On the other hand, the batch size for SARS-CoV-2 CT-scan dataset, COVID-CT dataset, and MOSMED dataset are taken as 50, 25, and 30 respectively. The graphs showing the final classification accuracies achieved after using different combinations of optimizers and learning rates on all three datasets are illustrated in Fig. 7.

It is to be mentioned that the accuracies reported in the plots are achieved after applying the FS algorithm, not the accuracies obtained by the deep learning models. Other deep learning hyperparameters such as momentum, regularization constant, etc. have been fixed to their standard values.

**Figure 8:**
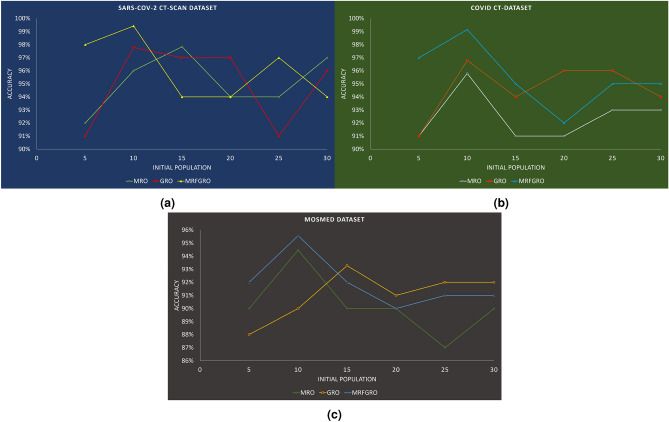
Graph showing the variation of classification accuracies with respect to various hyperparameters of proposed MRFGRO algorithm obtained on: (**a**) SARS-CoV-2 CT-Scan dataset, (**b**) Covid CT-dataset and (**c**) Mosmed dataset.

Some most important hyperparameters of MRFGRO based FS algorithm are the initial population, different kernel functions and regularization parameters of the SVM classifier. The variation of resultant accuracy concerning the initial population in all three datasets is given by Fig. 8.

The maximum accuracy for all three datasets is obtained with the initial population size of 10. Therefore, the initial population is fixed to 10 in this current study.

### Comparison with other optimization algorithms

To confirm the superiority of the MRFGRO algorithm, we have evaluated many popular optimization algorithms on all three datasets and compared the results with the results obtained by the MRFGRO algorithm. The algorithms which we have chosen for comparison are Genetic Algorithm (GA)^41^, Harmony Search Algorithm (HSA)^42^, Particle Swarm Optimizer (PSO)^43^, Atom Search Optimizer (ASO)^44^, Equilibrium Optimizer(EO)^45^, GRO and MRO. In addition to these, some hybrid algorithms such as GA+EO, PSO+ASO and HAS+GRO which gave good results are also reported in here. It is to be noted that, there are numerous optimization algorithms used for feature selections have been developed over past three decades. Therefore it is not possible to estimate performances of every possible combinations of these feature selection algorithms. Hence, from the aforementioned chosen algorithms, those combination which gave comparatively good and promising results are reported hereby.These wrapper based optimization algorithms have not been chosen on a random basis. It is to be noted that GA, HSA and PSO are very old algorithms with successful usage history in varied domains, whereas the other three are developed in recent times and have better efficiencies in many competent fields. The classification accuracies obtained by different optimization algorithms (used for FS in the literature) are shown in Table 5.

**Table 5:** Performance comparison of the proposed MRFGRO based FS algorithm with some popular FS algorithms.

Optimization algorithm	SARS-CoV-2 CT-scan dataset		COVID-CT dataset		MOSMED dataset	
	No. of features	Accuracy (%)	No. of features	Accuracy (%)	No. of features	Accuracy (%)
GA	942	92.43	779	91.11	802	91.19
PSO	739	90.15	855	94.49	864	93.29
HAS	1011	94.17	814	92.23	743	92.29
ASO	898	97.57	957	95.59	601	91.11
EO	917	96.69	913	96.28	698	90.19
GRO	868	97.79	809	95.79	713	93.28
MRO	997	97.84	877	96.78	759	94.47
GA+EO	942	95.48	779	95.28	789	94.21
PSO+ASO	1007	97.84	885	92.31	728	91.37
HAS+GRO	941	95.24	855	95.48	738	91.27
MRFGRO	**875**	**99.42**	**756**	**99.15**	**612**	**95.57**

Best accuracies and number of features selected corresponding to those accuracies are given in bold.

Proposed MRFGRO algorithm performs much better than the old and new FS algorithms considered here for comparison in terms of classification accuracy for all three datasets. Along with impressive classification accuracy, the number of features selected is also very less for the MRFGRO algorithm. This indicates that the MRFGRO algorithm is very efficient in selecting optimal features, thereby improving the overall classification accuracy.

### Comparison with recent methods

To gauge the goodness of the proposed framework, results obtained by some recent works on the aforementioned datasets have been compared with the results obtained by the present one. The results of the comparative studies are reported in Tables 6, 7 and 8. The proposed method achieves the best results over all the aforesaid datasets. Apart from that Shaban et al.^46^ with traditional machine learning with FS achieves impressive results of 96% in COVID-CT dataset. Whereas H. Aishazly^47^ by transfer learning with ResNet101 reports 99.4% accuracy on SARS-CoV-2 CT-scan dataset, which is almost the same as the achieved accuracy of MRFGRO model (99.42%). MOSMED dataset is not much explored so far. Rohila et al.^48^ did segmentation and classification, and reported 94.9% classification accuracy with their proposed ReCOV-101 net. As a whole, we can say that the proposed model of optimizing deep features using the MRFGRO algorithm outperforms all the models published recently for COVID-19 detection.

**Table 6:** Comparison of the proposed method with some state-of-the-art methods on COVID-CT dataset.

Work Ref.	Feature	Method of classification	Obtained accuracy (%)
Loey et al.^49^	Deep features	Data augmentation with classical augmentation technique and CGAN	82.91
Sakagianni et al.^50^	NA	AutoML Cloud Version	88.31
Jhao et al.^32^	Pre-trained CNN learns by itself	TL by DenseNet161 + CSSL	89.1
Alshazly et al.^47^	Transfer learning	DenseNet201	92.2
Shaban et al.^46^	GLCM	HFSM and KNN classifier	96
Saeedi et al.^21^	Deep features of DenseNet121	Nu-SVM	90.61 ± 5
Proposed algorithm	Deep features of ResNet18 and GoogLeNet	MRFGRO based FS algorithm	99.15

**Table 7:** Comparison of our proposed work with some state-of-the-art works on SARS-CoV-2 CT-Scan dataset.

Work ref.	Feature	Method of classification	Obtained accuracy (%)
Soares et al.^22^	Ensemble learning and classification	Adaboost	95.16
Jaiswal et al.^23^	Deep neural network learns relevant features by itself	DenseNet201	96.25
Alshazly et al.^47^	Transfer learning	ResNet101	99.4
Panwar et al.^51^	Deep neural architecture	Grad-CAM	95.61
Soares et al.^22^	Automated classification with deep xDNN	xDNN	97.38
Proposed algorithm	Deep features of ResNet18 and GoogLeNet	MRFGRO based FS algorithm	99.42

**Table 8:** Comparison of our proposed work with some state-of-the-art works on MOSMED dataset.

Work ref.	Feature	Method of classification	Obtained accuracy (%)
Sharma et al.^52^	No traditional features as it is an end to end learning method	ResNet18 + GradCAM	91
Rohila et al.^48^	Segmentation and classification	ReCOV-101	94.9
Proposed algorithm	Deep features of ResNet18 and GoogLeNet	MRFGRO based FS algorithm	95.57

## Conclusion

In this work, we have proposed a new hybrid FS model, called MRFGRO, which has been evaluated on three standard CT-scan based COVID-19 detection datasets. We have computed deep features instead of using traditional feature engineering in accomplishing this task, due to the advantages of deep features over traditional features as mentioned earlier. The state-of-the-art results obtained over all three datasets are reported in “Results and discussion” section. The effectiveness and superiority of hybrid MRFGRO over other FS algorithms are also provided in “Results and discussion” section. In spite of having many advantages of the proposed framework, there are some limitations too. Hereby we conclude our paper by mentioning some future extension of this work keeping in mind the limitations of the MFRGRO algorithm:

- We have evaluated our model on only CT-scan datasets. However, to confirm the robustness of the work, chest X-Ray image datasets can also be taken into consideration.
- Hyperparameters of transfer learning such as optimizer, learning rates, batch size etc. are very important for proficient learning of the CNN models. In this study, we have chosen the optimal parameters by performing some exhaustive experimentation. However, there are some efficient ways to find them, such as using some optimization techniques. Bayesian optimization can be used for hyperparameter fixing of deep learning models.
- In recent times, some advanced neural nets are also developed such as Squeeze net, Exception net, Capsule net, and so on. These nets can also be used for deep feature extraction.
- Initial population selection of MRFGRO algorithm can also be thought of which may help to increase the convergence rate of the said algorithm.

## Data Availability

No datasets are generated during the current study. The datasets analyzed during this work are made publicly available in this published article. These datasets can also be accessed via following links. SARS-Cov-2 CT-Scan dataset: https://www.kaggle.com/plameneduardo/sarscov2-ctscan-dataset; COVID-CT dataset: https://github.com/UCSD-AI4H/COVID-CT; MOSMED dataset: https://mosmed.ai/datasets/covid19_1110/.
